# Atypical Presentation of Meningococcemia as Gastroenteritis in an Adult: A Case Report

**DOI:** 10.7759/cureus.92126

**Published:** 2025-09-12

**Authors:** Usamah Al-Anbagi, Abdulrahman Saad, Tarek Ibrahim, Abdulqadir J Nashwan

**Affiliations:** 1 Internal Medicine, Hazm Mebaireek General Hospital/Hamad Medical Corporation, Doha, QAT; 2 Medicine, Ministry of Public Health, Doha, QAT; 3 Pharmacy, Hamad Medical Corporation, Doha, QAT; 4 Nursing and Midwifery Research, Hamad Medical Corporation, Doha, QAT

**Keywords:** atypical presentation, case report, gastrointestinal symptoms, meningococcemia, neisseria meningitidis, serogroup w135

## Abstract

Meningococcal disease is a life-threatening infection that can present with diverse and sometimes atypical manifestations, making early diagnosis challenging. We report the case of a 29-year-old man who presented with acute gastrointestinal symptoms, raising suspicion for gastroenteritis. Blood cultures later identified *Neisseria meningitidis* serogroup W135, confirming a diagnosis of meningococcemia. Unlike the typical presentation of meningococcal disease in adults, which often features fever, hypotension, myalgias, and a characteristic petechial rash, this patient exhibited predominantly gastrointestinal manifestations without neurologic signs or rash, leading to an early diagnostic challenge. Prompt recognition and empirical antibiotic treatment resulted in rapid clinical improvement and prevention of complications. This case underscores the importance of maintaining a high index of suspicion for meningococcemia in adults presenting with sepsis, even in the absence of classic features, as early intervention remains critical to improving outcomes.

## Introduction

Meningococcal disease, caused by Neisseria meningitidis, continues to be a major public health concern worldwide. It most frequently affects children and young adults, often leading to meningitis or septicemia. In the United States, annual incidence ranges from 0.5 to 1.5 cases per 100,000, with the highest rates seen in children under two years of age. Despite advances in antibiotic therapy and critical care, mortality remains between 10% and 15%, and rapid progression from mild illness to fulminant sepsis is not uncommon [1-3]. The classic presentation in adults includes sudden onset of fever, chills, myalgias, headache, hypotension, and a petechial or purpuric rash, with or without signs of meningeal irritation.

However, it is increasingly recognized that some meningococcal strains, particularly serogroup W, may present with prominent gastrointestinal symptoms such as abdominal pain, vomiting, and diarrhea, sometimes in the absence of a classic rash or meningeal signs. Recent epidemiological data indicate that serogroup W infections are more likely than other serogroups to initially mimic common gastrointestinal illness, which can obscure the diagnosis, delay appropriate therapy, and worsen clinical outcomes [4].

We present a case of meningococcemia in a previously healthy adult who initially exhibited only gastrointestinal complaints, highlighting the diagnostic pitfalls and emphasizing the need for early recognition and empirical management even when the clinical picture appears atypical.

## Case presentation

History

A 29-year-old man, previously healthy with no known comorbidities, presented with a one-day history of diarrhea, vomiting, and abdominal pain. He reported approximately 13 episodes of watery, yellowish stools since the onset, without blood or mucus. The diarrhea was accompanied by colicky abdominal pain localized to the umbilical region. He also developed four episodes of vomiting, along with a continuous low-grade fever. He typically consumes home-cooked meals, and none of his close contacts reported similar symptoms. There was no history of jaundice, dysphagia, or dyspepsia. He is a non-smoker, does not consume alcohol, and denies any history of recent travel or antibiotic use.

Examination

On examination, the patient was fully conscious, oriented, lying in bed, and appeared ill. He was not jaundiced or pale but showed signs of dehydration. There was no lymphadenopathy, no lower limb edema, and no skin rash, purpura, or meningeal signs. His vital signs were as follows: temperature 36.6 °C, heart rate 106 beats per minute, respiratory rate 22 breaths per minute, blood pressure 104/69 mmHg, and SpO₂ 99% on room air. Abdominal examination revealed a soft abdomen with slight tenderness over the umbilical area, no palpable masses or organomegaly, and normal bowel sounds. Cardiovascular and respiratory examinations were unremarkable. Neurologically, there was no neck stiffness, cognitive function was intact, cranial nerves were normal, and motor strength, sensation, and coordination were preserved.

Management

Initial laboratory investigations revealed markedly elevated white blood cell count, high urea and creatinine, low magnesium and phosphorus levels, elevated total bilirubin with a mixed pattern, and very high CRP and procalcitonin (Table 1).

**Table 1:** Laboratory Investigations MCV: Mean Corpuscular Volume; MCH: Mean Corpuscular Hemoglobin; ALT: Alanine Aminotransferase; AST: Aspartate Aminotransferase.

Parameters	On admission	On discharge	Reference values
Total leukocytes	49.6	13.5	(6.2 x10^3/uL)
Hematocrit	43.7	40.5	(40-50%)
Hemoglobin	14.9	13.1	(13-17 gm/dL)
MCV	87.4	91.5	(83-101 fL)
MCH	29.8	29.5	(27-32 pg)
Platelet	248	234	(150-410 x10^3/uL)
Serum urea	10.2	2.8	(2.5-7.8 mmol/L)
Serum creatinine	206	68	(62-106 umol/L)
Serum potassium	3.8	3.8	(3.5-5.3 mmol/L)
Serum sodium	136	139	(133-146 mmol/L)
Serum calcium	2.39	2.46	(2.2-2.6 mmol/L)
Serum total protein	83	67	(60-80 gm/L)
Serum albumin	33	25.4	(35-50 gm/L)
ALT	26	18.1	(0-41 IU/L)
AST	27	30	(0-41 IU/L)
Alkaline phosphatase	71	54	(40–129 IU/L)
Serum total bilirubin	39	9.3	(0-21 umol/L)
Serum magnesium	0.59	0.7	(0.7-1 mmol/L)
Serum phosphorous	0.77	0.75	(0.8-1.5 mmol/L)
Serum Direct bilirubin	24	4	(0-5 umol/L) (0.8-1.5 mmol/L)
C difficile toxin	Negative	-	Negative

**Figure 1:**
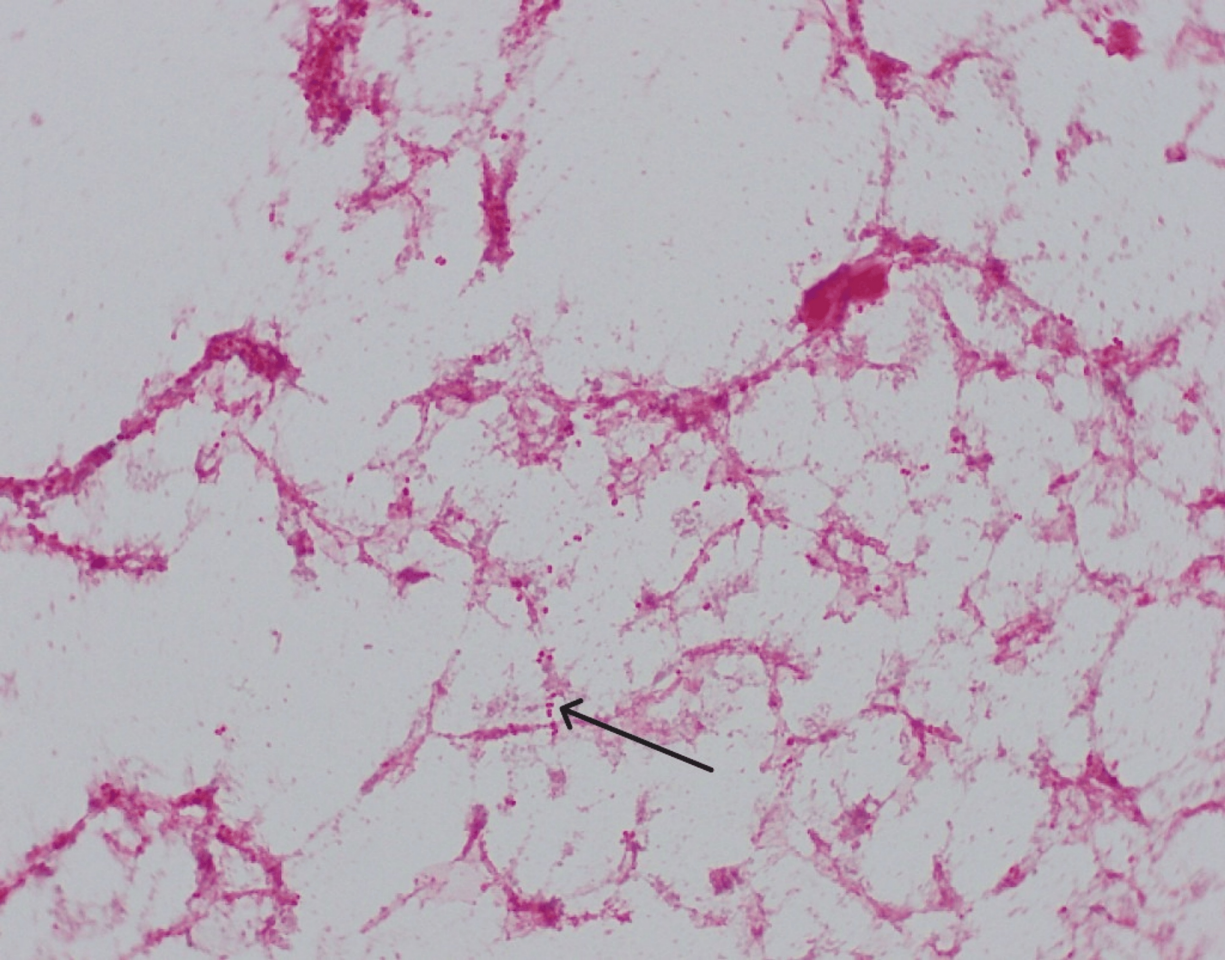
Gram stain of the blood culture revealed positive diplococci, black arrow

He was admitted as a case of gastroenteritis with sepsis, and a septic workup was performed, including two sets of blood cultures (aerobic and anaerobic), urine culture, and stool cultures. Empirical therapy with intravenous ceftriaxone 2 g daily was initiated alongside IV fluid resuscitation and electrolyte correction. By the second day, the patient showed clinical and laboratory parameters. On the third day, blood cultures turned positive for Neisseria meningitidis Group W135 (Figure 1), while stool cultures were negative, confirming a diagnosis of meningococcemia.

The organism was susceptible to ceftriaxone, and after discussion with the infectious disease team, it was decided to continue the same antibiotic and repeat blood cultures after 72 hours, which confirmed the blood cultures were sterile. The patient was subsequently discharged in very good condition with oral antibiotics to complete a total of 10 days of therapy, and a follow-up appointment was arranged. 

## Discussion

Neisseria meningitidis is a leading cause of bacterial meningitis, particularly in children and young adults, and ranks second among causes of community-acquired adult bacterial meningitis. In the U.S., invasive disease rates vary from 0.5 to 1.5 per 100,000, with the highest incidence in children under two [5,6]. Mortality averages around 10-15% [1-3]. Transmission occurs mainly through respiratory droplets, facilitated by overcrowding, poor sanitation, and malnutrition, though bacterial virulence and gaps in herd immunity also play roles [7]. Host factors like nasopharyngeal carriage, complement deficiencies, or use of complement-inhibiting drugs such as eculizumab increase individual risk [8].

Our case diverges significantly from the classic presentation of meningococcemia, which typically manifests in adults as a sudden onset of fever, chills, myalgia, headaches, hypotension, and a characteristic petechial or purpuric rash; meningismus may also develop [2,5]. In contrast, this patient presented almost exclusively with gastrointestinal symptoms - diarrhea, vomiting, and abdominal pain - initially mimicking acute gastroenteritis without evident rash or neck stiffness. Such presentations are distinctly atypical and pose a significant diagnostic challenge, especially in adults. The literature indicates that gastrointestinal symptoms, such as nausea and diarrhea, are more commonly reported in invasive meningococcal infections caused by serogroup W. These features remain uncommon in adults and often overshadow more classic signs [2,4].

Several case reports have similarly documented management of meningococcemia in adults who initially presented with gastrointestinal symptoms. For example, a case series described adult patients admitted with presumed gastroenteritis or lower respiratory tract infection, whose blood cultures later grew Neisseria meningitidis, and who recovered fully following appropriate antibiotic therapy [6]. These cases reinforce the notion that invasive meningococcal disease can masquerade as common, benign conditions and still carry the risk of rapid deterioration if not treated emergently.

The diagnostic delay seen in our case highlights the critical need for clinicians to maintain a high index of suspicion for meningococcemia in any adult presenting with sepsis alongside gastrointestinal symptoms. Empiric administration of antibiotics is vital; guidelines emphasize initiation of appropriate therapy, ideally within the first hour of suspected meningococcal disease, to improve clinical outcomes. Our patient’s favorable recovery following early empirical ceftriaxone, which was later confirmed to be sensitive, underscores how prompt recognition and early initiation of antibiotics, even in the context of an atypical presentation, can alter the disease trajectory.

Meningococcal disease can range from mild fever and bacteremia to overwhelming infection that progresses to death within hours. It most often presents as meningitis alone, meningitis with sepsis, or meningococcemia without obvious meningeal involvement [2,9]. Early symptoms typically include sudden fever, headache, nausea, vomiting, and intense muscle aches, often more severe than seen in viral infections. Sometimes a preceding nonsuppurative pharyngitis leads to initial misdiagnosis, but the rapid worsening usually points away from simple respiratory tract infection. Complications like shock, disseminated intravascular coagulation, and purpura fulminans can arise quickly. Immune-mediated issues such as arthritis, pleurisy, vasculitis, and pericarditis may also appear, often later in recovery [10].

Prompt antibiotic therapy is essential in suspected meningococcal disease to improve survival, particularly in patients with sepsis, petechiae, or signs of meningitis [2,11]. Empirical treatment usually begins with ceftriaxone, favored over penicillin for its reliable coverage, ease of dosing, and ability to clear nasopharyngeal carriage. Once culture and sensitivity results arrive, therapy may be narrowed; if the isolate is fully penicillin-susceptible, either ceftriaxone or high-dose penicillin G is appropriate [12]. In severe cases complicated by purpura fulminans, protein C concentrate may help correct coagulation abnormalities [13]. Droplet precautions should be maintained for at least 24 hours after starting antibiotics to limit transmission. Close contacts need rapid chemoprophylaxis, ideally within 24 hours of exposure and no later than two weeks, using rifampin, ciprofloxacin, or ceftriaxone, with local antibiogram [14].

Before antibiotics, meningococcal infections were almost always fatal, with mortality up to 90% [15]. Sulfonamides and later penicillin greatly improved survival, yet deaths still occur in 10-15% of cases today despite critical care [16]. Worse outcomes are linked to bleeding, neurological signs, older age, and delays in antibiotics. Outbreak-related infections also show higher fatality than isolated cases. Interestingly, some studies suggest women may have higher mortality, especially with meningitis, though reasons are unclear [2,17]. These patterns highlight how bacterial serotypes, patient factors, and early treatment together shape prognosis.

## Conclusions

This case illustrates how meningococcemia can present in adults with non-specific gastrointestinal symptoms, lacking the hallmark signs of meningitis or rash that usually heighten clinical suspicion. Such atypical presentations underscore the necessity of considering invasive meningococcal infection in any patient who appears septic, regardless of initial symptom focus. Maintaining vigilance for this potentially fatal disease and initiating empiric antibiotics without delay remain key to improving patient survival. Ultimately, awareness of these uncommon presentations can prevent missed or delayed diagnoses and ensure timely, life-saving interventions.

## References

[1] Cohn AC, MacNeil JR, Clark TA (2013). Prevention and control of meningococcal disease: recommendations of the Advisory Committee on Immunization Practices (ACIP). MMWR Recomm Rep.

[2] Al-Anbagi U, Al Maslamani M, Al-Khal A (2025). A cluster of meningococcal serogroup W135 infections: a case series. Cureus.

[3] MacNeil JR, Blain AE, Wang X, Cohn AC (2018). Current epidemiology and trends in meningococcal disease—United States, 1996-2015. Clin Infect Dis.

[4] Bertrand-Gerentes I, Fanchon L, Coste F, Glover RE, Guiddir T, Taha MK (2023). Range of clinical manifestations caused by invasive meningococcal disease due to serogroup W: a systematic review. Infect Dis Ther.

[5] Stinson C, Burman C, Presa J, Abalos M (2020). Atypical presentation of invasive meningococcal disease caused by serogroup W meningococci. Epidemiol Infect.

[6] Farooq M (2019). Atypical presentation of Neisseria meningitidis. Dubai Med J.

[7] Glover JA (1918). The cerebrospinal fever epidemic of 1917 at "X" depot. Epidemiol Infect.

[8] Applegate AO, Fong VC, Tardivel K, Lippold SA, Zarate S (2016). Notes from the field: meningococcal disease in an international traveler on eculizumab therapy—United States, 2015. MMWR Morb Mortal Wkly Rep.

[9] Heckenberg SG, de Gans J, Brouwer MC (2008). Clinical features, outcome, and meningococcal genotype in 258 adults with meningococcal meningitis: a prospective cohort study. Medicine (Baltimore).

[10] Feigin RD, Dodge PR (1976). Bacterial meningitis: newer concepts of pathophysiology and neurologic sequelae. Pediatr Clin North Am.

[11] Barquet N, Domingo P, Caylà JA (1997). Prognostic factors in meningococcal disease: development of a bedside predictive model and scoring system. Barcelona Meningococcal Disease Surveillance Group. JAMA.

[12] McNamara LA, Potts C, Blain AE (2020). Detection of ciprofloxacin-resistant, β-lactamase-producing Neisseria meningitidis serogroup Y isolates—United States, 2019-2020. MMWR Morb Mortal Wkly Rep.

[13] Faust SN, Levin M, Harrison OB (2001). Dysfunction of endothelial protein C activation in severe meningococcal sepsis. N Engl J Med.

[14] Gardner P (2006). Clinical practice. Prevention of meningococcal disease. N Engl J Med.

[15] Flexner S (1913). The results of the serum treatment in thirteen hundred cases of epidemic meningitis. J Exp Med.

[16] Mbaeyi SA, Bozio CH, Duffy J, Rubin LG, Hariri S, Stephens DS, MacNeil JR (2020). Meningococcal vaccination: recommendations of the Advisory Committee on Immunization Practices, United States, 2020. MMWR Recomm Rep.

[17] Bloch D, Murray K, Peterson E (2018). Sex difference in meningococcal disease mortality, New York City, 2008-2016. Clin Infect Dis.

